# Findings from a mixed-methods evaluation of a multi-level adolescent and youth reproductive and maternal health intervention in Karnali Province, Nepal

**DOI:** 10.1186/s12905-023-02425-w

**Published:** 2023-05-17

**Authors:** Rajan Bhandari, Sara Malakoff, Dipendra Singh Thakuri, Roma Balami, Sangita Khatri, Callie Simon, Wendy Castro, Nana Apenem Hanson-Hall

**Affiliations:** 1 Save the Children International, Kathmandu, Nepal; 2 Independent Contractor, Bogota, Colombia; 3 Save the Children US, Washington, DC USA; 4 University Research Co.,LLC, Formerly With Save the Children US, Washington, DC USA

**Keywords:** Healthy transitions, Adolescent girls and young women, Reproductive maternal and neonatal health, Socio-ecological model

## Abstract

**Introduction:**

Adolescent girls and young women (AGYW) in Nepal have disproportionately poor reproductive and maternal health outcomes. In response, Save the Children, the Nepal government, and local partners designed and implemented Healthy Transitions for Nepali Youth, a multi-level integrated intervention. The intervention aimed to improve reproductive, maternal, and newborn health knowledge, attitudes, and behaviors among AGYW, and address gender attitudes and norms in four districts of Karnali Province, Nepal.

**Methods:**

Married and unmarried AGYW aged 15–24 were engaged in a small group, curriculum-based intervention; husbands and families received home visits, using short videos to catalyze discussion; communities were engaged through dialogue-based activities; and the health system was made more adolescent-responsive through quality assessments, training, and supervision. An external organization conducted a quantitative survey with a sample of 786 AGYW intervention participants at baseline and 565 of the same AGYW at endline. Pooled linear regressions were estimated for each indicator to assess the statistical significance of differences between baseline and endline. Focus group discussions and key informant interviews were conducted with AGYW, husbands, families, community leadership, and program implementers. Data analysis was done through STATA 14^th^ version and NVivo.

**Results:**

The percentage of AGYW currently using a modern contraceptive method increased significantly, and more AGYW believed that their family was supportive of delaying marriage and motherhood at the endline. Young women’s knowledge of danger signs during labor increased, and there was a significant improvement in essential newborn care practices immediately after birth. AGYW reported shifts towards more gender equitable attitudes and behaviors, including related to decision-making about reproductive and maternal health.

**Conclusion:**

Positive shifts in reproductive, maternal, and newborn health and gender knowledge, attitudes, and behavior were observed among AGYW, their male partners, and families. The results can inform the design of future interventions to effectively reach this critical population.

**Trail registration:**

Not applicable.

## Background

In Nepal, adolescents and youth make up more than one third of the total population with 24.2% of the population between the ages of 10–19 and a further 8.9% of the population between the ages of 20–24 [1]. While 43% of married women of reproductive age in Nepal are currently using a modern contraceptive method, only 15% of married adolescents 15–19 are using a modern method. Nearly one fifth (17%) of Nepali adolescent girls have begun childbearing by the age of 19, with stark differences by level of education and wealth quintile [2].

Karnali Province (formerly Province 6) in the hill region of Nepal has the second youngest median age at marriage (17.4) and first birth (19.8) in Nepal [2]. The Province also has the second highest total fertility rate (2.8), and second highest adolescent childbearing rate in the country; of young women ages 15–19 in Karnali Province, 19% have begun childbearing [2]. Less than half of women in the Province (47.3%) receive antenatal care (ANC) during all specified months as recommended by the Nepali Ministry of Health (4, 6, 8 and 9 months), and it has the highest number of women who report not receiving any ANC, over twice as high as any other province at 14.7% [2]. Karnali Province similarly has a lower skilled birth attendance rate than the national average (35.3%) [2]. Improving the ability of adolescent girls and young women (AGYW) ages 15–24 to choose when, and if to become pregnant, and to seek care during and after pregnancy has the potential to contribute significantly to reproductive, maternal, and newborn health (RMNH) outcomes in Karnali Province. In addition, investments in improving the health outcomes of adolescents can, in turn, improve the ability of adolescent girls and young women to complete their education and participate more actively in the economic well-being of their families and communities. In response to this opportunity, Save the Children, together with 4 local non-governmental organization partners (NGOs), and 9 municipal/local governments, designed and implemented the Healthy Transitions for Nepali Youth Project (Healthy Transitions) in four districts of Karnali Province. Local partners included Social Awareness Center (SAC) Nepal, Surkhet; Karnali Integrated Rural Development and Research Center (KIRDARC), Kalikot; Everest Club (EC), Dailekh; and Panch Tara Yuwa Samrakshak Manch (PTYSM), Jajarkot.

### Program description

Healthy Transitions (May 2018-September 2021) aimed to improve the RMNH of AGYW ages 15-24 and their babies in four districts of Karnali Province: Jarjakot, Surkhet, Dailekh, and Kalikot. Healthy Transitions worked in ten geographical clusters in each district, covering 40 health facilities and their catchment areas. Healthy Transitions was designed through a collaborative process with contributions from Save the Children technical staff with expertise in RMNH for AGYW, the Government of Nepal, and local partners. Drawing on a review of evidence of AGYW RMNH interventions and recommended practices, the socio-ecological model was selected as the theoretical underpinning and primary design framework for Healthy Transitions [3, 4]. In addition to the socio-ecological framework, the design of Healthy Transitions incorporated three theory-based approaches demonstrated to contribute to effective RMNH programming for AGYW: 1) the interventions aimed to be gender-transformative [5]; 2) the interventions were gender-synchronized [6]; and 3) interventions were tailored to the specific age, life-stage, and unique characteristics of AGYW, their husbands, and their families [7–9]. Using the socio-ecological framework and the three key approaches, mutually reinforcing intervention strategies were selected for each level of the socio-ecological model (individual, family/interpersonal, community and system level). Interventions were designed to engage girls and young women at the individual level, their male partners and families at the interpersonal level, their communities, and the health system. The selection and design of each intervention drew on evidence-based practice from the literature [8–11] as well as the learning and tools developed and tested by Save the Children in Nepal, including My First Baby [12] and the Fertility Awareness for Community Transformation Project, which included the Pragati Games [13] (interactive activities and discussion prompts for improving fertility awareness and raising social norms around fertility and family planning). Healthy Transitions aimed to achieve four objectives – one at each level of the socio-ecological model – that, together, contribute to the goal of improving RMNH among AGYW:

1. AGYW have improved knowledge and capabilities to delay and space pregnancies, care for themselves and their baby during pregnancy and after childbirth, and participate in household financial management.
2. Husbands of married AGYW have more gender-equitable attitudes, and support RMNH care-seeking behaviors among AGYW.
3. Families and community members are supportive of more equitable household gender norms and of RMNH care-seeking behaviors among AGYW.
4. High-quality RMNH services are available and responsive to the needs of adolescents and youth.

### AGYW interventions at individual level

To engage AGYW, the project used a small group, “safe spaces” approach [14]. Local partners formed 360 groups of AGYW composed of 18–22 members each in January 2019. Healthy Transitions was designed to include 2-cohorts of girls, each cohort going through the program for 12 months. The evaluation described in this paper focuses on Cohort 1, which included 8,755 girls. The second cohort started shortly before the COVID-19 pandemic in February 2020 and received an abbreviated intervention (about ten months) of group-based sessions and curriculum-based radio drama that covered all content, which reached 6,708 AGYW in Cohort 2. Overall, Healthy Transitions reached 70.08% of 22,064 total AGYW in the catchment areas.

Young mentors aged 20–24 years, who were local women hired by the project, recruited participants and facilitated the curriculum for each group. Social mobilizers were recruited to supervise and provide technical support to mentors. Each female social mobilizer supervised the work of 3 mentors. Mentors and social mobilizers received 7 days of training. Group sessions lasting 45–60 min were conducted fortnightly amongst the same group of participants to complete 24 sessions over 12 months from February 2019 through January 2020 for Cohort 1. The *Swastha Rupantaran* (Healthy Transitions) curriculum included 17 sessions on RMNH, 5 sessions on violence, mental health and self-efficacy, and 2 sessions on financial literacy. Discussion of gender (in)equality as a key driver of RMNH, violence, self-efficacy, mental health, and financial literacy was woven throughout the sessions. The group sessions also included Pragati games, adapted to create a safe space to allow for in-depth discussion and reflection on social and gender norms [13]. Each AGYW group visited the health facility of their catchment area to enable them to become more knowledgeable, familiar, and linked with the strengthened adolescent responsive RMNH services offered.

### Interventions at family and interpersonal level

The family level intervention involved visits by social mobilizers to the homes of married AGYW to engage male partners, young couples, parents and in-laws in dialogue using a tablet-based job aid, approved by the Ministry of Health and Population, National Health Education Information Communication Centre (NHEICC). The job aid was comprised of 6 short videos that addressed: 1) gender equality in the household; 2) spousal engagement in newborn and maternal care; 3) mother-in-law support for pregnant women during a husband’s absence due to migration; 4) couple FP conversations after childbirth; 5) participation of women in household decision making; 6) support for girls’ education. Each social mobilizer was intended to conduct 20 home visits per month, including monthly visits to each enrolled participant’s household. The social mobilizers were unable to conduct all intended home visits due to capacity constraints and difficult terrain. Ultimately, 7,155 Cohort 1 individuals were reached by a home visit and 6,243 from Cohort 2.

### Interventions at community level

Several interventions took place within the community to generate dialogue and support for more equitable household gender norms, and to promote RMNH care-seeking behavior among AGYW. Trained social mobilizers conducted community dialogues 4-times per month using the NHEICC-approved community dialogue and reflection tool and Pragati games. Other events, such as sports, celebrations, quiz or game shows, and street-drama performances, were held approximately once every 6 months, and upon request by municipal/local governments. All the community events aimed to spur communities to reflect on their attitudes and beliefs, to support more gender equitable norms, and to provide support to AGYW in seeking information and services related to RMNH. These social events were timed with migration patterns, so that activities were held when men were likely to be present.

### Adolescent- and youth- responsive health system strengthening

Healthy Transitions, in partnership with the Rural Municipalities, Municipalities and PNGOs, conducted adolescent and youth responsive RMNH needs assessments in the 40 health facilities in the project intervention area. The assessments examined RMNH service availability, quality of services provided to adolescents and youth, staffing, equipment, commodity availability, and infrastructure. Skills-based training was provided by certified trainers from the National Health Training Center (NHTC) to nursing staff (auxiliary nurse midwives, staff nurses), paramedics (auxiliary health workers, health assistant) and adolescent health focal persons based on findings of the assessments. Training topics included: comprehensive FP counseling and provision (including provision of long-acting methods), Adolescent Sexual and Reproductive Health, skilled birth attendant, Community-Based Integrated Management of Newborn and Childhood Illness, and how to use the District Health Information System. Project staff and provincial health officials followed up with trained providers during joint monitoring and supervision visits to assess progress at least once per quarter. In addition, certified trainers conducted ongoing supportive supervision and onsite coaching 1–2 times per year to reinforce knowledge and skills. To enable adolescents and youth to provide feedback on the quality of services and promote youth-led social accountability for health services, Healthy Transitions supported the annual use of Save the Children’s Partnership Defined Quality for Youth process [15, 16].

## Methods

### Baseline data collection

IMPAQ LLC and its Nepal-based data collection partner, Solutions, evaluated the first cohort of Healthy Transitions using a mixed methods pre-post design to assess changes in outcomes for the same project beneficiaries over the course of 1 year of project implementation. A quantitative survey was conducted in February 2019 with AGYW across 40 health catchment areas of the 4 districts (Table 1). The survey employed a two-stage sampling strategy to select a sample of young women aged 15 to 24, married and unmarried, from among those who had enrolled in the small group component of Healthy Transitions.

**Table 1:** Baseline and endline quantitative interview sample of AGYW (15–24), by district

Districts	Baseline			Endline		
	**Unmarried**	**Married**	**Total**	**Unmarried**	**Married**	**Total**
Kalikot	129 (65%)	69 (35%)	198	100(67%)	50(33%)	150
Jarjakot	142 (68%)	66 (32%)	208	86(62%)	53(38%)	139
Dailekh	99 (55%)	81 (45%)	180	73 (56%)	58(44%)	131
Surkhet	126 (63%)	74 (37%)	200	94(65%)	51(35%)	145
Total	496 (63%)	290 (38%)	786	353 (63%)	212 (37%)	565

First, two of the mentors (small group facilitators) were chosen randomly in each site (total of 80), and then ten young women per mentor were selected for the survey (total of 800). 786 AGYW were interviewed during the February 2019, with nearly 2% non-response. The survey was intended to measure baseline levels of RMNH knowledge and practices, gender-related attitudes and behaviors, self-efficacy, and financial literacy. Sample size was determined based on an estimated minimum detectable effect of 12 percentage point difference between treatment and control for a binary outcome, assuming attrition of 20%, power of 80%, and significance level of 0.05.

Healthy Transitions complemented the quantitative baseline study with qualitative methods to provide deeper understanding of participants’ views and experiences. A total of 31 focus group discussions (FGDs) led by 2 female field supervisors (one lead and one note taker affiliated with the research agency) in all 4 districts were conducted at baseline using a semi-structured guide to further explore young women’s autonomy, the role their family members play in RMNH decisions, and emotional well-being as AGYW transition through major life events. FGDs were held with 4 groups each of married (*n* = 35) and unmarried young women (*n* = 29), 7 groups of husbands (*n* = 51), 8 groups of mothers-in-law (*n* = 52), and 8 groups of influential community members (*n* = 33), including Health Facility Operation and Management Committee members (committees that operate and manage health facilities and ensure service quality). With consent, FGDs were recorded. At the close of each session, facilitators summarized main points using a summary form. To ensure data quality, IMPAC LLC and Solutions worked closely with fieldwork managers and supervisors on a regular basis in the field to oversee data quality and to provide teams with technical assistance.

### Endline data collection

The endline study tracked changes in knowledge, attitudes, and behaviors for 565 of the same AGYW surveyed at baseline, a 72% response rate **(**Table 1**)**. The evaluation team was unable to reach the remaining AGYW surveyed at baseline because they had migrated for marriage, further study, or work. An attrition analysis was conducted to determine potential bias in the endline sample. For the qualitative data collection, the project selected sites in each district that reflected the diversity of its project area and conducted a total of 24 FGDs, 4 FGDs each with: unmarried AGYW, married AGYW, mothers-in-law, husbands, social mobilizers, and mentors (Table 2). Key informant interviews (KIIs) were conducted with 21 project implementers, 8 local government leaders, and 21 health staffs with the assistance of district coordinators.

**Table 2:** Endline qualitative focus group discussion sample, by district

Respondent Type		Number of Participants per FGD			
		Dailekh	Jajarkot	Kalikot	Surkhet
Participants	Unmarried AGYW	8	8	11	10
	Married AGYW	7	8	10	10
	Mothers-in-law	9	8	8	7
	Husbands	6	8	8	5
Implementers	Social Mobilizers	5	4	3	5
	Mentors	7	6	8	8

### Data analysis

Following completion of field activities, data collectors conducted a final review of the survey data to clean it and check for duplicates, skip pattern logic, and for completeness. Survey responses were compiled into a master file for analysis. For quantitative data, descriptive analyses were conducted to determine the socio-demographic characteristics of respondents. Pooled linear regressions were estimated for each indicator to assess the statistical significance of differences between baseline and endline.

To finalize the qualitative data, the note taker and lead reviewed the notes for the FGDs and KIIs, using the recordings to verify data, identify patterns, and extract relevant quotes. The English translation of Nepali transcripts was done by the research team on the same day of the interview. Identifying information was removed from the notes, retaining only district and respondent type. The notes were then compiled into an Excel template for analysis. Thematic framework containing central themes and subthemes was prepared. The verbatim and quotations were used to demonstrate the perception of the participants. The transcription files were uploaded in the NVivo to aggregate responses, highlight any differences by district or respondent type, and to synthesize the major themes across the interviews and FGDs. The team triangulated the qualitative analysis with the quantitative survey findings.

## Results

The attrition analysis revealed that the overall sample at endline had similar characteristics, as measured at baseline, to the sample that was not surveyed at endline. The data presented in Table 3 shows the subgroup composition of AGYW surveyed and not surveyed at endline.

**Table 3:** Baseline characteristics of AGYW survey respondents, by attrition

	**Surveyed at Endline** **[*N* = 565]**		**Not Surveyed at Endline** **[*N* = 221]**	
	**N**	**%**	**N**	**%**
***District***				
Dailekh	131	23%	49	22%
Jajarkot	139	25%	69	31%
Kalikot	150	27%	48	22%
Surkhet	145	26%	55	25%
***Age Group***				
15–19	389	69%	135	61%**
20–24	176	31%	86	39%**
***Marriage***				
Never Married	350	62%	133	60%
Ever Married	215	36%	88	40%
***Parity***				
Never given birth	400	71%	149	67%
1 birth	85	15%	28	13%
2 + births	80	14%	44	20%
***School Status***				
In School	375	66%	129	58%**
Out of School	190	34%	92	42%**
***Education***				
Less than grade 8	179	32%	72	33%
Grade 8 or above	386	68%	149	67%
***Caste***				
Brahmin/Chhetri	320	57%	123	56%
Dalit	170	30%	70	32%
Other	75	13%	28	13%
***Wealth***				
Lowest	187	33%	85	38%
Middle	224	40%	69	31%*
Highest	154	27%	67	30%

^*^p < 0.10; ** *p* < 0.05; *** *p* < 0.01

The majority of the 565 AGYW surveyed at endline were 15–19 years of age (69%). Over half of the sample (57%) were from the Brahmin/Chhetri group (Table 3). The majority of the AGYW were still in school (66%) but there was variation according to marital status, of 215 ever married (210 currently married) AGYW, only 13.8% were enrolled in school.

### Exposure to healthy transitions

Seventy-five percent of AGYW surveyed at endline attended all, or nearly all, of the 24 group sessions; very few attended fewer than half of the sessions. AGYW from poorer households were more likely to have attended fewer sessions. As noted above, social mobilizers conducted fewer home visit sessions than planned. No family received the goal of all 6 visits. Of 210 currently married AGYW in the sample, only 36% reported a home visit; about half (51%) of those reported only one visit.

### Support for delaying marriage and childbearing

**Fig. 1:**
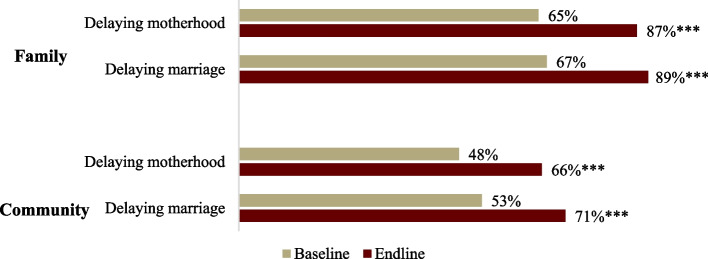
Support for delaying marriage and motherhood until age 20 among AGYW

In Nepal, the legal age of marriage is 20 years. At the endline, 89% of AGYW strongly agreed that their family and community were supportive of delaying marriage until age 20, and 87% believed that their family and community was supportive of delaying motherhood, both significant, positive changes from baseline (Fig. 1). In the majority of focus groups where this topic was broached, participants mentioned that no one should marry before the age of 20. Still, of the 10 AGYW who married over the year of implementation, the median age of marriage was 18.

*There used to be the practice of getting married at a young age. After participating in the program, we know that youths should not get married at a young age. Thus, youths get married only after they turn 20 years old. —Mothers-in-law, FGD*

### Family planning knowledge, attitudes, and use

**Fig. 2:**
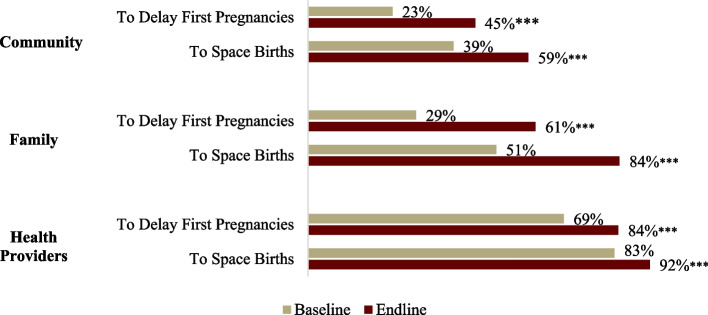
AGYW’s perception of community, family, and health provider support for FP use to delay first pregnancy and space births

AGYW knowledge of all methods of contraception increased significantly (*p* < 0.01). The largest increases were in knowledge of intrauterine contraceptive device (IUCD), lactational amenorrhea method (LAM), emergency contraception, and standard days methods. In addition, respondents were significantly more likely at endline to report that their families, communities, and health providers supported FP use to delay first pregnancy and to space subsequent births (*p* < 0.01). Across all groups, responses indicated greater support to use FP to space pregnancies rather than to delay first births (Fig. 2).

*When women give birth to a daughter, their families coerce women to give birth sooner, even after being advised by mentors to wait 2 to 4 years. The family prefer sons, so women end up giving birth to many children. —*Mentors, FGD

**Fig. 3:**
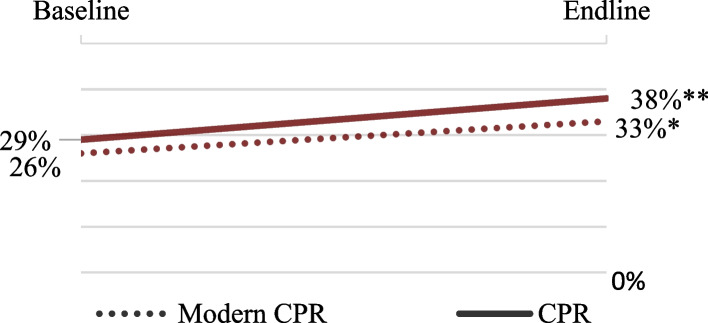
Change in contraceptive prevalence rate and modern contraceptive prevalence rate among ever married AGYW from baseline to endline

Analysis of contraceptive use was limited to ever-married women. Median age at first sexual intercourse for women aligns with median age at first marriage in Nepal, indicating that, for women, sex is most often initiated within the context of marriage. For the 212 ever married women in the endline sample, the modern CPR was 33%, significantly increased from 26% at baseline (Fig. 3).

At endline, 48% of married AGYW currently using any contraceptive method reported using injectables, with 16% using implants, 15% withdrawal, 9% condoms, 9% oral contraceptive pills, 2% IUCD, and 1% emergency contraception. There was no significant difference in the types of contraceptive methods used between baseline and endline. The qualitative data suggests that the high percentage relying on withdrawal could be related to the large number of husbands who migrate for work, causing some AGYW to be embarrassed or uncomfortable accessing contraception due to stigma.

All women (100%) who obtained a method at a health facility were very satisfied with the way they were treated by the provider, a significant 38-percentage point increase from baseline, where only 62% were very satisfied with their treatment (*p* < 0.001).

*We used to be shy to ask for family planning methods and contraceptives, but now we can easily talk to the health practitioners at the health center*. —AGYW, FGD

### Antenatal care (ANC) knowledge and service use

Routine ANC and the ability to recognize danger signs during pregnancy are critical to timely and appropriate decisions that can reduce maternal mortality and morbidity. AGYW named on average 2.6 out of 5 danger signs during pregnancy at endline, up from 1.8 at baseline (*p* < 0.015). Over half identified severe headache, dizziness, blurred vision, swollen hands and face, and extreme lower abdominal pain to be danger signs. Less than half recognized vaginal bleeding and stiff limbs/seizure/headache as danger signs, although the number still significantly increased from baseline among both married and unmarried respondents.

AGYW sampled at baseline universally agreed that women should receive ANC during pregnancy. At endline, participants were asked how many visits a pregnant woman should have, and about the timing of these visits. Ninety percent (90%) of AGYW said that women should have four or more ANC visits. Of the 41 AGYW in the sample who gave birth within the year, all reported receiving ANC during pregnancy, and 95% reported receiving four or more visits. The findings of this survey were found to be consistent with the perception and practices of the FGD participants.

*Regular checkups, at least four times, is a must during pregnancy*. —Married women, FGD

*Before, during pregnancy, our wives used to go for health checkups on her own. Now, we go with her for moral support*. —Husbands, FGD

### Labor and delivery knowledge and service use

AGYW surveyed had varying degrees of input into decision-making around birth. Thirty-five of the 41 AGYW who gave birth between baseline and endline (85%) reported having a say in the decision about where to give birth, 27 (66%) of whom said that this was a joint decision with her husband. Mothers-in-law were involved in the decision for 16 of the women (39%). Six women (15%) indicated that they were not part of the decision-making process regarding care during pregnancy and had no say around where they would deliver.

Of 41 women who gave birth over the course of the year between baseline and endline, 78% delivered in a health facility (compared to 71% of 237 women who had given birth at baseline). There was no skilled birth attendant (SBA) present for eight out of the nine births that took place at home.

**Fig. 4:**
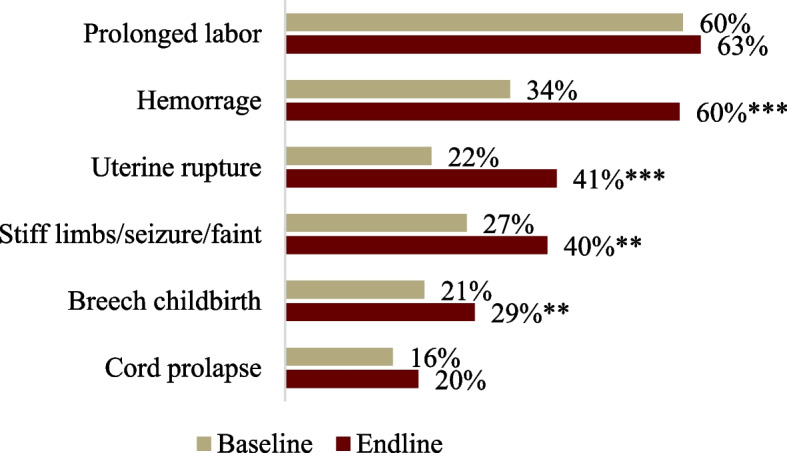
Changes in knowledge of danger signs during labor among AGYW from baseline to endline

Knowledge of danger signs during labor increased significantly, from 1.8 at baseline to 2.5 out of 6 at endline (Fig. 4). While married AGYW could identify more danger signs than unmarried (2.7 compared to 2.4 respectively), both showed significant increases in knowledge.

*The health facility staff are able to provide better services after receiving training [through Healthy Transitions]. Before, health staff could only save the mother during complications. However, after the SBA sessions, there are instances when they have become successful in saving both the mother and the child*. —Social mobilizers, KII

### Postnatal and newborn care knowledge and service use

Similar to ANC and health facility delivery, virtually all AGYW expressed positive attitudes about postnatal care (PNC) at both baseline and endline, agreeing that newborns should be vaccinated, receive regular checkups from a health provider, and that women should likewise have postnatal checkups. Data from the FGDs affirmed these findings. Participants spoke of new mothers receiving better treatment, including increased access to nutritious food, keeping warm, avoiding strenuous chores like lifting heavy loads, and being encouraged to take more rest post-birth.

*Women who had not gone for a single checkup for their past deliveries have started to go to health centers on a regular basis [after giving birth]. They eat nutritious food, and new mothers are told to rest*. —Social Mobilizers, KII

**Fig. 5:**
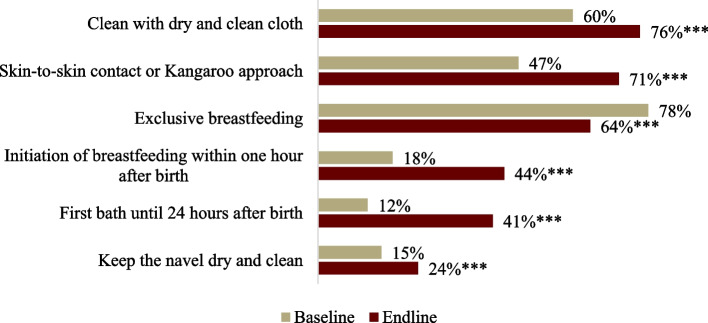
Changes in knowledge of essential newborn care among AGYW from baseline to endline

There were significant changes in AGYW’s knowledge across 5 of 6 components of essential care. Given the universal support of exclusive breastfeeding for at least six months, and the significant increase in those who know to initiate breastfeeding within one hour of birth, we believe that this finding is flawed (Fig. 5). Similarly, at endline, AGYW on average named 3.2 practices out of five, a significant increase from 2.3 at baseline. The qualitative findings also agree with these changes in the community.

*In the past, there was a tradition of bathing a newborn immediately after birth. However, now we know that we should wait at least 24 hours after the birth to bathe the child. —* Mothers-in-law, FGD

Regarding danger signs for newborns, AGYW could identify significantly more signs of danger at endline compared to baseline, 2.8 versus 2 respectively.

### Gender attitudes and behaviors

AGYW were asked whether they agree or disagree with a series of statements pertaining to common gendered beliefs, behaviors, and norms. There were large changes towards more gender equitable attitudes and behaviors from baseline to endline among AGYW, for example 23% points fewer agreed that her primary role is to cook for the family and take care of the home (Table 4).

**Table 4:** Distribution (%) of AGYW gendered attitudes at baseline and endline

Do you agree that:	Baseline (*N* = 786)	Endline (*N* = 565)
Men need sex more than women do	66%	53%***
A woman cannot refuse to have sex with her husband	55%	39%***
A woman should tolerate violence in order to keep her family together	45%	26%***
A woman’s most important role is to take care of her home and cook for her family	48%	25%***
There are times when a woman deserves to be beaten	42%	25%***
It is a woman’s responsibility to avoid getting pregnant	37%	24%***
If a married couple is unable to have children, there is something wrong with the wife	17%	14%
Men should have the final say in all family matters	21%	13%***
A man with only daughters is unfortunate	12%	6%***
Not having a son reflects bad karma and lack of moral virtue	12%	5%***
It is more important for men to get an education than women	11%	5%***
Men who migrate for labor can experiment with pleasures, as long as he fulfills his financial obligations to his family	9%	5%***
It is acceptable for a young woman to drop out of school to marry	3%	1%*

^*^p < 0.10, ** *p* < 0.05, *** *p* < 0.01

Some participants of FGD remarked the women have begun to play a more active role in their household’s financial affairs. Examples cited of women’s participation in household finances include opening bank accounts. However, some of the social mobilizers highlighted that gender attitudes toward the son preference and pressure for women to bear a son remain deeply rooted in the community.

*If the first born is a daughter, the families take care of both the child and the mother, but if the woman already has a daughter, they are not given as much care. [If the second birth] is a son, this is celebrated. —Social Mobilizers, KII*

## Discussion

AGYW reported significant positive changes in their RMNH knowledge, behavior and gender attitudes after participation in Healthy Transitions for 12 months. Qualitative findings from husbands, families, and community leaders reinforce these reported changes. We acknowledge that these results are from an evaluation without a comparison group, and as such cannot be interpreted as having necessarily been caused by Healthy Transitions (see *Limitations*). However, the magnitude and consistent positive direction of the quantitative findings and the complementary qualitative findings suggest that Healthy Transitions is a promising intervention.

Theory, evidence, and implementation experience suggests that one reason Healthy Transitions may have contributed to positive changes in RMNH and gender outcomes is that it employed gender-transformative interventions at each level of the socio-ecological model. In this way, Healthy Transitions aligns with, and reinforces recommendations from across AGYW literature, including Temin and Heck’s review of community-based girl groups [14] and Levy et al. review of successful programs targeting gender inequality and restrictive gender norms for the health and wellbeing of children, adolescents, and young adults [17, 18].

In addition to the use of gender-transformative interventions at each level of the socio-ecological model, Healthy Transitions was unique in that it addressed the integrated family planning/reproductive health (FP/RH), MNH, gender, and financial literacy needs and priorities of AGYW. In particular, MNH has not been traditionally included in AGYW programming. Linking FP/RH to MNH for AGYW responds to the context of early marriage and childbearing, by supporting AGYW to choose when, and if, to have a baby, and supporting healthier outcomes for mothers and babies [19].

While the findings show positive changes over the period of intervention around childbearing, sex discrimination in childhood opportunities, and the role of women in the home, strong societal preference and pressure for women to bear a son remain and continue to constrain the reproductive health and rights of AGYW in Karnali Province and elsewhere in Nepal. For example, the qualitative findings demonstrate a continued son preference that makes it difficult for AGYW to space births, especially if their first child is a girl. Despite improvements from baseline to endline, AGYW still perceive relatively limited support for delaying marriage and pregnancy from their community, and do not enjoy full autonomy in their RMNH care-seeking behavior. Addressing entrenched community norms and attitudes likely requires adaptations to program approaches and longer implementation of key approaches at scale. Two key adaptations that could strengthen the Healthy Transitions approach include: 1) addressing more thoroughly the context of male migration, and 2) increasing the intensity of the home visit intervention to further catalyze normative change at the household and community level. In terms of male migration, while the CPR significantly increased among survey respondents, 15% of married AGYW were relying on withdrawal. Qualitative data highlighted how this could be linked to perceptions that contraception was unneeded due to a husband’s absence, combined with stigma around accessing contraception while a marital partner was away. We echo previous literature that has called for tailoring programs to the specific needs of those who face spousal migration, as this affects not only timing and exposure to project interventions, but also impacts the conversation couples have around contraceptive use, and desired fertility/reproductive intentions [19–22]. Future adaptations may also engage boys and young men at earlier ages, before they begin work that may propel them to migrate. Regarding the intensity of home visits, Healthy Transitions planned to have more such visits to engage married men, couples, and in-laws. These home visits were designed to be participatory, using videos to support reflection, and to challenge gender norms at household and community level. The expected number of home visits was not completed. An increased number of home visits may have supported further shifts in husband and family support for AGYW as well as shifts in underlying social and gender norms. Evidence is not conclusive on the optimal dose for changes in behaviors and norms [10], but some projects have found greater degree of FP/RH behavior and norm change with higher numbers of home visits. For example, the similar PRACHAR program in Bihar, India found 7- 12 visits as the “tipping point” where more than half of young women contraceptive users had initiated use, [9] whereas only 36% of AGYW in the endline evaluation of Healthy Transitions reported receiving any home visit at all.

Finally, in an effort to position the key approaches for scale-up and sustainability, Healthy Transitions was designed jointly with government and local partners, and all project materials were approved by the relevant government agency, NHEICC. There are some early signs of sustainability, for example AGYW groups have transitioned to community-based mothers’ groups, registered with local governments, and some of these governments like Gurvakot and Bheriganga Municipalities in Surkhet district have allocated budgets for groups to continue sessions and other awareness activities. In addition, they have included key Healthy Transitions approaches in their workplans and budgets. That said, with the exception of the health system strengthening interventions, the Healthy Transitions interventions are designed to be implemented by community workers and local partners. To be sustained and scaled, the government of Nepal, and others, would need to fund these local partner and community-led efforts, or approaches would need to be adapted to be implemented through existing government cadres.

### Limitations

These positive findings should be interpreted with several limitations in mind. As noted above, a randomized control trial or quasi-experimental study with a comparison group was not feasible given implementation and budgetary constraints. Another limitation of the study is that the sample size was set at 80% power to detect an overall effect of the intervention, which did not allow for analysis of effect among different subgroups. The sample was drawn from AGYW who self-selected into the Healthy Transitions program. Those AGYW are generally more educated than the average population and the Brahmin/Chhetri castes are slightly over-represented. However, 70.08% of the AGYW in the intervention communities enrolled in the program so it does reflect a majority of AGYW in these communities. As a result of the selection bias/sampling, the findings cannot necessarily be extrapolated to women with different characteristics. We relied on self-reported data, which may be biased toward socially desirable answers. We utilized methods to reduce bias (such as employing experienced all-female enumerators to ask sensitive questions), but still suspect that AGYW did not always disclose information they deemed sensitive. Finally, the simultaneous sequencing of qualitative and quantitative data collection efforts did not allow for additional follow-up on the decline in breastfeeding knowledge. Future qualitative exploration and other studies on breastfeeding knowledge and practices may provide additional useful insights for implementers.

## Conclusion

Evidence from the first cohort of AGYW who participated in Healthy Transitions demonstrates that using a robust socio-ecological approach, encompassing integrated and mutually-reinforcing activities at the individual, family, community and health facility level, while robustly addressing underlying gender attitudes and behaviors at each level, yielded promising results. The mixed methods evaluation showed that Healthy Transitions positively influenced knowledge, attitudes and behaviors related to RMNH care-seeking in the four districts, along with indicators of gender equitable attitudes that affect the health and well-being of AGYW. Future programs and approaches for AGYW in Nepal and elsewhere can draw on the learning and promising practices from Healthy Transitions.

## Data Availability

The datasets generated and analysed during this study will be available from the corresponding author on reasonable request.

## Abbreviations

AGYW: Adolescent Girls and Young Women
ANC: Antenatal Care
CPR: Contraceptive Prevalence Rate
FGD: Focus Group Discussions
FP: Family Planning
HTNYP: Healthy Transitions for Nepali Youth Project
IUCD: Intrauterine Contraceptive Device
KII: Key Informants Interview
LAM: Lactational Amenorrhea Method
NHEICC: National Health Education Information Communication Centre
NHTC: National Health Training Centre
PNC: Postnatal Care
RMNCH: Reproductive, Maternal, and Newborn Health
SBA: Skilled Birth Attendant
